# Blockchain Based Secure Federated Learning With Local Differential Privacy and Incentivization

**DOI:** 10.1109/tp.2024.3487819

**Published:** 2024-11-08

**Authors:** SAPTARSHI DE CHAUDHURY, LIKHITH REDDY MORREDDIGARI, MATTA VARUN, TIRTHANKAR SENGUPTA, SANDIP CHAKRABORTY, SHAMIK SURAL, JAIDEEP VAIDYA, VIJAYALAKSHMI ATLURI

**Affiliations:** 1 Department of Computer Science and Engineering, Indian Institute of Technology, Kharagpur 721302, India; 2 Management Science and Information Systems Department, Rutgers University, Newark, NJ 07102-3122 USA

**Keywords:** Encrypted model parameter, federated learning, HyperLedger fabric, local differential privacy, session key

## Abstract

Interest in supporting Federated Learning (FL) using blockchains has grown significantly in recent years. However, restricting access to the trained models only to actively participating nodes remains a challenge even today. To address this concern, we propose a methodology that incentivizes model parameter sharing in an FL setup under Local Differential Privacy (LDP). The nodes that share less obfuscated data under LDP are awarded higher quantum of tokens, which they can later use to obtain session keys for accessing encrypted model parameters updated by the server. If one or more of the nodes do not contribute to the learning process by sharing their data, or share only highly perturbed data, they earn less number of tokens. As a result, such nodes may not be able to read the new global model parameters if required. Local parameter sharing and updating of global parameters are done using the distributed ledger of a permissioned blockchain, namely HyperLedger Fabric (HLF). Being a blockchain-based approach, the risk of a single point of failure is also mitigated. Appropriate chaincodes, which are smart contracts in the HLF framework, have been developed for implementing the proposed methodology. Results of an extensive set of experiments firmly establish the feasibility of our approach.

## I. INTRODUCTION

The goal of Federated Learning (FL) is to dynamically train a machine learning (ML) model through participation of independent but collaborating clients (interchangeably called nodes) [1]. A central entity designated as the server commences the learning process by selecting a model and setting its initial parameters. Over multiple rounds, the nodes train the model with their local datasets, send the updated gradients to the server, and receive the newly computed global weights from the server. Model training stops once convergence is achieved or after a certain maximum number of rounds have been completed [2], [3]. However, the standard implementations of FL are susceptible to various kinds of security and privacy attacks, such as data [4] and model poisoning [5], [6], interference [7] and Byzantine attacks [8], model extraction attacks [9], evasion attacks [10], etc. This happens particularly because the updated model data is shared across the clients.

To guard against inference and other forms of privacy attacks, FL is combined with Local Differential Privacy (LDP) [11], [12]. In the resulting LDP-FL technique, the nodes obfuscate their gradients using a privacy parameter ϵ before sharing the same with the server. While LDP-FL addresses the problem of privacy leakage, it cannot guard against server failure, which can have a catastrophic impact on the entire FL process [13], [14], [15], [16]. Blockchains have been introduced to provide a more robust approach to FL wherein a well formed smart contract acts as the server and the clients constitute the nodes of the blockchain [17]. It makes FL inherently fault tolerant and attack resilient since the same set of transactions is available to all the peer nodes of the blockchain after consensus-based validation. Another challenge in widespread adoption of FL in general, and LDP-FL in particular, is an inherent lack of commitment from the nodes in sharing their model parameters. This is primarily because there is no incentivization framework that would encourage the nodes to do so. For example, even if a node obfuscates its parameters to a great extent by choosing a small value of ϵ (and hence, higher privacy), it can still access the same updated model as any other node that uses a higher value of ϵ.

In a recent work [18], we attempted to address the above mentioned challenges of FL by combining LDP-FL with incentivization in a permissioned blockchain framework [15]. The approach named as BTLF (*Blockchain-based TBI-LDP-FL*), uses a token-based incentivization (TBI) mechanism in which participating nodes receive tokens if they share their model parameters. Such tokens can subsequently be used for accessing global model weights. The number of tokens awarded is in proportion to the value of ϵ so that the nodes are encouraged not to excessively obfuscate the data they share, beyond their own privacy requirement. While the approach does alleviate some of the concerns identified in the literature, it also has its own dependencies. The threat model assumed in [18] is that the nodes behave non-maliciously and appropriately deduct tokens while passing on updated model weights to their clients. Such an assumption may not hold in real life scenarios where the permissioned blockchain nodes are authenticated but not necessarily trusted.

Considering the above limitation of the previous work, in this paper, we propose a novel cryptographically protected methodology that encrypts updated weights using a session key for each FL round. The key is shared with the clients only after deducting an appropriate quantum of tokens, thereby ensuring fair use of incentives. A set of well-formed chaincodes handle all the operations, thus ensuring correctness and integrity of the system. We have implemented the proposed approach named SBTLF (Secure-BTLF) using the HyperLedger Fabric-based permissioned blockchain framework and evaluated it extensively under different FL setups. Our evaluation indicates that the proposed approach of privacy-preserved FL framework through the cryptographically protected token-based incentivization mechanism converges well with the desired accuracy while introducing minimal overhead on the clients for additional memory usage. The main contributions of this paper are summarized below.

1. We propose a token-based incentivization mechanism for federated learning with local differential privacy in a permissioned blockchain setting that prevents non-malicious behavior from the participating nodes while passing the model parameters.
2. To alleviate the above shortcoming, we propose a novel cryptographically-protected methodology called SBTLF to encrypt the model parameters using a session key shared with the FL clients only after deducting an appropriate quantum of tokens. This step provides an added layer of security over our earlier work [18] to eradicate the impact of malicious nodes that mishandle the token deduction protocol.
3. We have implemented SBTLF using HyperLedger Fabric and tested it thoroughly under various scenarios. The proposed method ensures secure and privacy-preserving model training with marginal overhead regarding memory requirements for additional cryptographic computations.

The rest of the paper is organized as follows. Section II provides some background on federated learning, differential privacy, and blockchains. The proposed SBTLF framework is described in Section III. Details of HyperLedger Fabric-based implementation of SBTLF is presented in Section IV. Section V discusses the results of an extensive set of experiments carried out on an end-to-end working SBTLF system. Section VI reviews recent literature and we finally conclude with future directions for research in Section VII.

## II. PRELIMINARIES

This section introduces some of the basic concepts underlying the work presented later in the paper.

### A. FEDERATED LEARNING

Federated learning (FL) [1] orchestrates collaborative training of local deep neural network (DNN) models among N distributed clients connected to a central server. The process commences with the server randomly initializing the model parameters, denoted as $\mu_{o}$, which are then distributed to the clients to initialize their respective model copies. Each client independently trains its model using local dataset for multiple epochs, eventually producing updated model parameters, $\mu_{u}$. The process of federated learning in each round includes averaging all the $\mu_{u}$ ‘s received from the clients to obtain $\mu_{\text{fed}}$ (the parameters of the global model). Such an iterative process, known as a federation round, continues until $\mu_{\text{fed}}$ either converges or a predetermined number of rounds is completed [19]. It has been shown that $\mu_{\text{fed}}$ produces almost the same accuracy as that of a local model trained on the complete data [20].

It is well known that collection and analysis of user data at scale is a driving force behind the recent progress in machine learning. However, data collected from users tend to be private and also sensitive. Further, the same can potentially be linked to other more confidential information. Owing to this, users are often sceptical about divulging their personal data, especially in the face of new and emerging technologies for in-depth data mining and analytics. To regulate the activities of corporate firms with respect to consumer data they have access to, various countries have enacted privacy laws such as General Data Protection Regulation (GDPR) [21] and California Consumer Privacy Act (CCPA) [22]. Therefore, privacy preservation when dealing with data has become an urgent issue that needs to be addressed [23]. Privacy concerns in the context of Federated Learning have been raised as well, especially due to its potential vulnerability to inference attacks [24].

### B. DIFFERENTIAL PRIVACY

Differential privacy is a mathematical framework for ensuring privacy of individuals in datasets, first proposed in [25]. It allows data to be analyzed without revealing sensitive information about any particular user in the dataset, thereby providing a strong guarantee of the individual’s privacy. According to their definition, the outcome of the mechanism should not be significantly affected by the presence or absence of any particular record in the dataset. Hence, for any two datasets that differ in only one record, if the probability of any outcome occurring is nearly the same, then the mechanism is considered to be differentially private.

**FIGURE 1.**
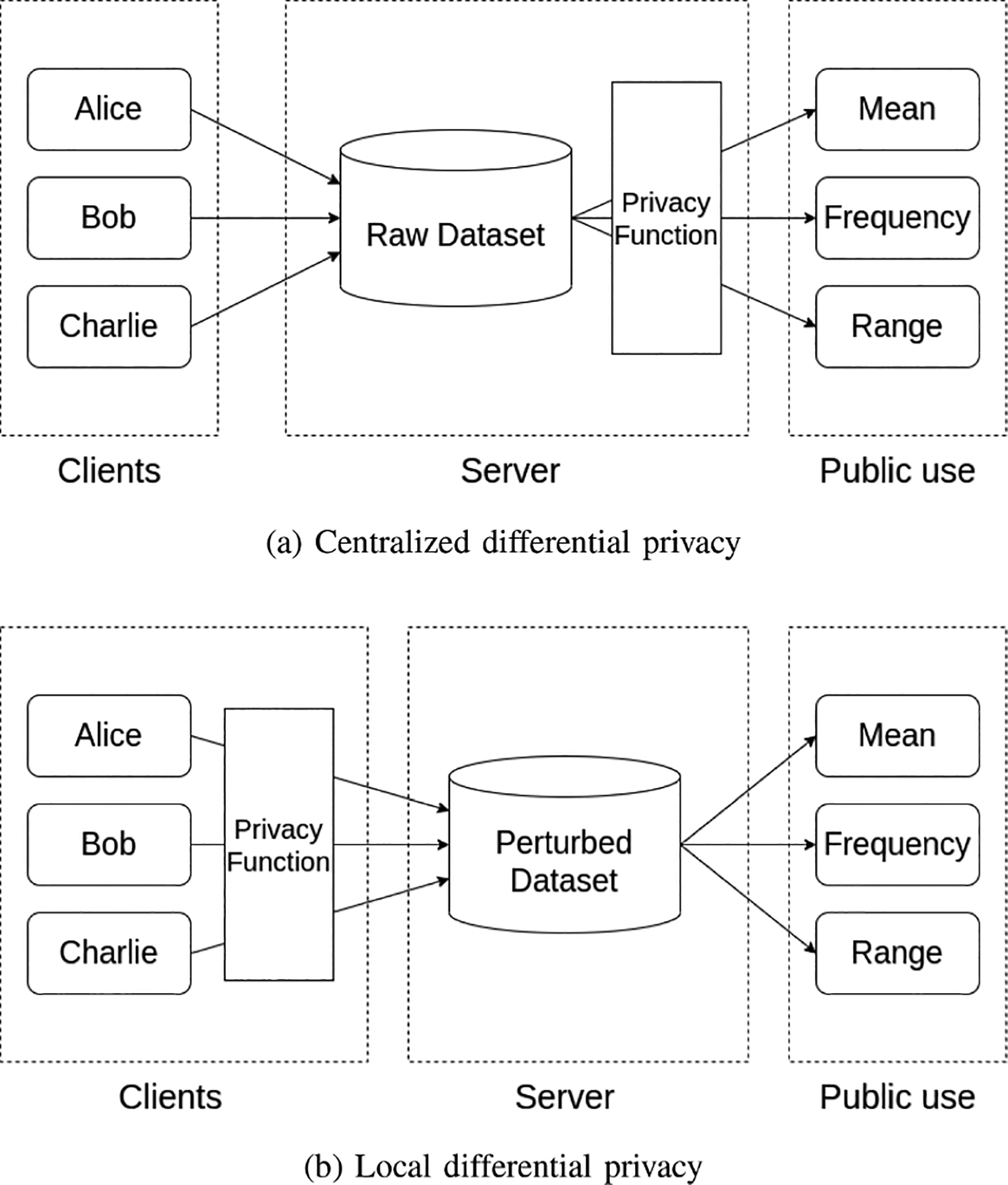
Two forms of differential privacy models - centralized and local.

In the traditional model of differential privacy, also known as centralized differential privacy (CDP), there exists a trusted aggregator or curator which collects and holds the sensitive data of all the individuals as shown in Fig. 1(a). It is responsible for protecting their privacy by adding a measured amount of noise, i.e., perturbing the data by infusing sufficient noise to the output. Effectively, the goal is to mask the contribution of individual data elements and yet preserve overall analysis accuracy. The aggregator, however, has to first collect original data of the users before releasing the perturbed aggregated information publicly [23].

A concern with CDP is that there is an implicit but complete trust on the data curator, which may not always be the situation in real world. Even the largest and most reputable companies cannot guarantee their customers’ privacy and may fall victim to data breaches. To address this problem, a mechanism called local differential privacy (LDP) has been proposed. In LDP, a user’s data is perturbed locally before it is sent to the aggregator (Fig. 1(b)). The original data is only accessible to the owner, which provides a much stronger privacy guarantee for the user. In the LDP model, a curator holds only a perturbed version of the data and not the original data. Also, all the training and any form of querying is performed on this perturbed dataset only. Thus, LDP protects against data disclosure even to the untrusted curators and relieves the burden on the trusted data curators to keep data secure [23]. LDP is often defined in terms of what is known as ϵ-Local Differential Privacy. As defined in [26], a randomized mechanism *M* is said to satisfy ϵ-Local Differential Privacy if and only if for any pair of input values *v* and $v^{\prime }\in D$ and for any possible output S⊆Range(ℳ),

(1) $$\text{Pr}[ℳ(v)\;\in \;S]\;\leq \;e^{\epsilon }\text{Pr}\left[ℳ\left(v^{\prime }\right)\;\in \;S\right]$$

Here ϵ>0 is the privacy budget; the smaller the value of ϵ, the stricter protection with lower data availability, and vice versa.

Federated Learning is a natural application domain for LDP and has been investigated from different aspects and application domains [27], [28], [29], [30], [31], [32]. Several relaxations of differential privacy also exist [33], [34], which could allow for a better privacy-utility trade off.

### C. PERMISSIONED BLOCKCHAINS AND HYPERLEDGER FABRIC

A blockchain is a decentralized append-only ledger, where the blocks are added by a set of distributed validators, who either mine a new block by solving a cryptography puzzle or generate and validate the new block through collective attestations. A block in a blockchain contains a set of ordered transactions, representing the execution steps of the world state of the system. Blockchain helps a set of participants, called nodes, execute transactions without explicitly trusting each other. The transactions become immutable once a block is added to the blockchain ledger. Considering the nature of participants, there are two possible types of blockchains – *open* or *permissionless* blockchain, where the participants do not need any authentication or pre-authorization to join the blockchain network, and *closed* or *permissioned* blockchain, where the participants are authorized and authenticated through some membership services.

HyperLedger Fabric (HLF) provides a modular and extensible open-source platform to deploy and operate permissioned blockchains. It is hosted by the Linux Foundation [35] and provides a modular language platform to implement smart contracts. The current state of the ledger is termed as the *World State* that represents the latest values of all committed transactions. *Organizations* in HLF are the entities that constitute the decentralized network. Each organization has its own peers with specific roles and permissions in the network. The *Peers* maintain the ledger and handles the state updated based on the transactions. They are responsible for endorsing, validating, and committing transactions in the underlying blockchain.

In HLF, the smart contracts are termed as *Chaincodes*, which implement the business logic governing the blockchain transactions. Chaincodes are executed on the HLF distributed ledger network and provide rules and agreements to govern how the data will be accessed, updated, and validated within the HLF network. An *Asset* in HLF is a physical or a digital entity having certain value which is owned or controlled by an organization or an individual. Assets can be tangible (e.g., real estate, vehicles) or intangible (e.g., intellectual property, patents), and can be represented digitally on the blockchain ledger. In HLF, transaction validation is done through *Endorsements*, where a specific member of endorsing peers validate and support the transactions before committing them to the ledger. The endorsement policies are configured to ensure security and reliability of the network by enforcing that the transactions meet the criteria set by the organizations.

Based on these preliminaries, we next describe the SBTLF framework.

## III. PROPOSED APPROACH

**FIGURE 2.**
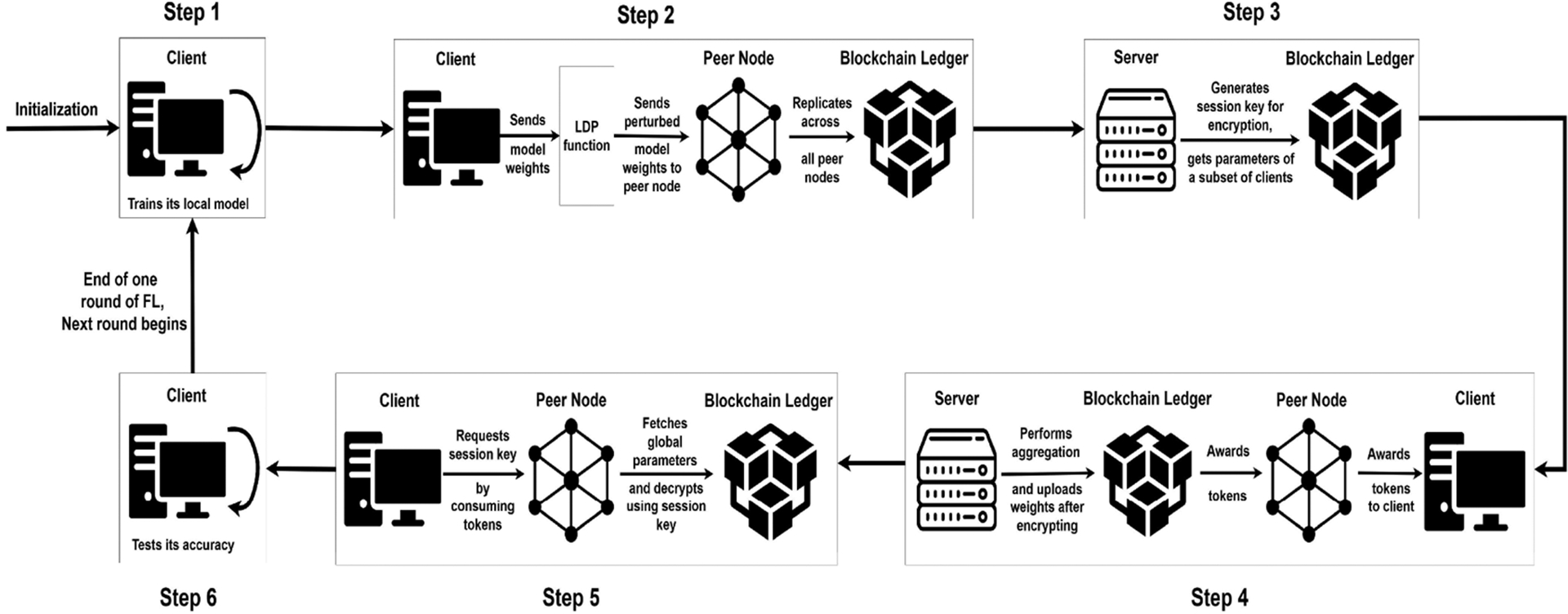
Overview of the SBTLF framework.

In this section, we outline our proposed approach SBTLF that integrates symmetric key cryptography and private data collections of HLF along with LDP and incentivization mechanisms for Federated Learning. Fig. 2 shows a high-level flow diagram of SBTLF.

### A. CREATION OF HLF NETWORK

Before the Federated Learning process commences, participating organizations collaborate to form a consortium within the HLF network. Each organization registers itself to the consortium and creates a private data collection between the central server and the organization. Within an organization, multiple peers may be enrolled. Additionally, a single peer can serve as an interface for interactions with multiple clients. However, each client must undergo registration and authorization processes before being able to submit transaction proposals.

**Algorithm 1:** Pseudo Code for Client.

1: (m,w)←init().
2: **while** till convergence or maxRounds reached **do**
3: w←trainModel(m,w)
4: $w^{\prime }\;\leftarrow \;\text{LDP}(w,\;\epsilon )$ ▷ Perturb local model parameters
5: $\text{putParameters}\left(w^{\prime },\;\epsilon \right)$
6: $w_{\text{enc}}\;\leftarrow \;\text{getEncryptedGlobalParams}(\text{round})$
7: requestSessionKey(round)
8: key←getSessionKey(round)
9: $w\;\leftarrow \;\text{decrypt}\left(w_{\text{enc}},\;\text{key}\right)$
10: **end while**

### B. BLOCKCHAIN-BASED LDP-FL

Once registered, each client follows the pseudo code given in Algorithm 1. Here, m represents the model and w the set of model weights. ϵ is the privacy budget of the particular client. w_enc denotes the encrypted model weights. Upon initiation, the client initializes a deep neural network (DNN) model with randomized parameters as shown in Line 1 of the algorithm (Step 1 of Fig. 2) Subsequently, this model undergoes training using the available local dataset for multiple epochs, resulting in updating of the model parameters. The updated model parameters are then subjected to perturbation, following the prescribed privacy factor ϵ of the LDP technique.^1^ The clients then submit their perturbed weights to the peers of the blockchain network to be securely written onto the HLF ledger (Step 2 of Fig. 2). Note that training is done entirely off chain on the local system of the client, and only the perturbed model weights are uploaded to the ledger via a chaincode.

**Algorithm 2:** Pseudo Code for Server.

1: **while** next round local parameters available **do**
2: $\mu_{u}\;\leftarrow \;\text{getLocalParameters}(n)$
3: aggregate←0
4: **for** $\mu_{u}$ in $\mu_{u}$ **do**
5: $\text{aggregate}\;\leftarrow \;\text{aggregate}+\mu_{u}[i]$
6: **end for**
7: $\mu_{\text{fed}}\;\leftarrow \;\frac{\text{aggregate}}{\text{num}}$
8: $\mu_{{\text{fed}}_{e}}=\text{symmetricEncrypt}\left(\mu_{\text{fed}},\;\text{key}\right)$
9: $\text{putEncryptedParams}\left(\mu_{{\text{fed}}_{e}}\right)$
10: clientKeyRequests ← getKeyRequests()
11: **for** org, round in clientKeyRequests **do**
12: putSymmetricKey(org, *round, key*_*round*_)
13: **end for**
14: **end while**

After writing the perturbed weights in the ledger, clients can submit a transaction through the peers for the encrypted global parameters. They make a request for the session key as shown in Line 7 of the algorithm by invoking the chaincode. After submitting the request, the client will get the session key, which can be used for decrypting the model parameters using symmetric key cryptography. This process continues until convergence is achieved or a maximum predefined number of rounds is reached.

Algorithm 2 shows the pseudo code followed by the server. Here, $\mu_{u}$ is the array of model weights received by the server from the clients and n is the number of clients whose parameters are being fetched by the server. $\mu_{\text{fed}}$ denotes the aggregated model weights calculated off-chain. *org* stands for the identifier of the organization which has requested the symmetric key for decryption and *round* represents the round number of the FL process for which the key has been requested.

The server gets a random subset of clients’ local model parameters from the ledger, which are aggregated off-chain as shown in Lines [3–7] of the algorithm. The aggregated parameters $\left(\mu_{\text{fed}}\right)$ are then encrypted using symmetric key encryption with a randomly generated key. This is the session key and is stored off-chain as metadata for the round (Step 3 of Fig. 2). The encrypted parameters $\left(\mu_{\text{fe}d_{e}}\right)$ are written to the ledger (Step 4 of Fig. 2). Writing encrypted global parameters ensures that no client can access the global parameters without obtaining the session key. When a client requests for a session key, tokens from its corresponding organization get deducted (Step 5 of Fig. 2). Section III-C discusses about the token incentivization in this setting. The clients can check the model accuracy by running it on its local dataset (Step 6 of Fig. 2).

The server periodically checks if there are any pending requests from clients for the session key. If there is any, the server writes the session key of the requested round in the private data collection of the requested client’s organization. This ensures that no one except the server and the requested client’s organization members will be able to access the session key, and hence, the global parameters updated in that round. This process continues until there are no more local parameters available.

### C. TOKEN-BASED INCENTIVIZATION

The method proposed in BTLF [18] involves assigning tokens to individual clients within an organization. Updates to these tokens are then performed on the specific client’s holding whenever relevant events occur. This method, however, could result in a scenario where different clients within the same organization possess varying numbers of tokens. To address this inconsistency, in SBTLF, we transition to an accreditation system where tokens are assigned to the organization as a whole rather than the individual clients. In this approach, any event triggering a token update, regardless of whether it is initiated by or for a specific client, modifies the organization’s token pool.

The events that update an organization’s tokens are as follows:

1. A client requests a session key for a round that has not been previously accessed by any client within the organization. A fixed number of tokens from the organization are deducted.
2. Server gets the client’s local parameters for a round. The number of tokens awarded (T) to the organization is as per (2).
(2) $$T=\sum_{\text{client}\in C}\;0.5+\frac{\epsilon_{\text{client}}-\epsilon_{\text{min}}}{2\left(\epsilon_{\text{max}}-\epsilon_{\text{min}}\right)}$$
  where C is the set of random clients of the organization selected for aggregating weights. $\epsilon_{\text{min}}$ and $\epsilon_{\text{max}}$ respectively denote the lowest and highest values of ϵ among those clients chosen by the server.

Any client within the organization has the ability to request for the session key of a specific round. Once obtained, all clients from that organization can utilize this key to decrypt global parameters. While clients could technically aggregate the local parameters off-chain, this process would demand a considerable amount of computing power, surpassing what a single server could manage when aggregating the local parameters. Consequently, clients are more inclined to engage in the FL process, utilizing tokens to participate, rather than individually aggregating all parameters off-chain.

It may further be argued that a client could potentially obtain the most updated weights without having contributed except in the initial and final phases to training. However, it would be difficult for any client to determine when the final round is going to happen since that requires it to read the weights for determining the accuracy level. Also, in order to guard against such a situation, values of tokens can be made to degrade over rounds. As a result, the clients will not be able to save their tokens for only a final round acquiring of model weights. Introducing this feature does not have any significant impact on the overall methodology.

## IV. IMPLEMENTATION DETAILS

In this section, we give a detailed description of our implementation of the SBTLF framework in HyperLedger Fabric, a modular and extensible open-source system specifically designed for deploying and operating permissioned blockchains [35]. In HLF, a *chaincode* is a collection of smart contracts, representing the business logic governing transactions within the network. It is run on the HLF distributed ledger network and facilitates execution of transactions. An *asset* in HLF refers to any digital or physical entity that has value and is owned or controlled by an organization or an individual. While there are several competing LDP-FL approaches available, the mechanism proposed in [31] has been used in our work due to its practical approaches towards supporting LDP-FL in real-life applications.

### A. CHAINCODE IMPLEMENTATION

Within an HLF network, each client and the server functions as a node in the HLF infrastructure. If a client or server’s organization is not yet enrolled in the network upon logging in, the enrollment process is initiated. After the organization enrollment is completed, the client or server can proceed to enroll and register as a member or participant within that specific organization. The client and server backend are developed using Node.js, with support for HLF, and the chaincodes are written in Golang.

The chaincode asset definition is as follows:


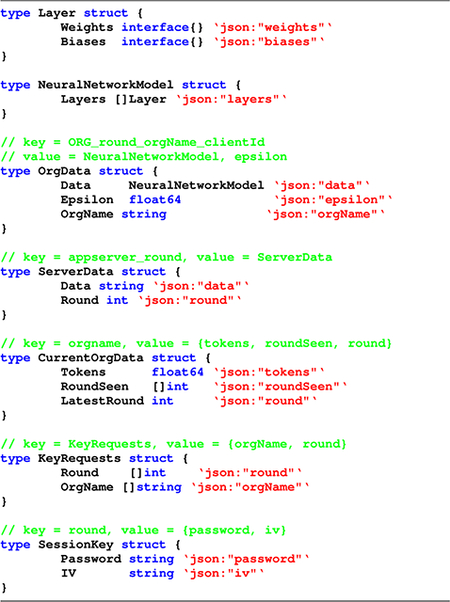


#### Layer struct:

It stores the a DNN layer parameters written into the ledger. The Weights and Biases can be of any dimensions, for supporting wide variety of neural networks.

#### NeuralNetworkModel struct:

It stores a list of layer parameters, so that each layer can be separately accessed.

#### OrgData struct:

It is used to store the complete data (the DNN model, privacy budget ϵ, organization name) for a particular round, organization, client. The key for accessing the data to the struct is *ORG_pl2x − amp − iso− lt;round > _pl2x − amp − iso − lt; orgName > _pl2x−amp − iso − lt; client Id* >. This will ensure that complete ledger data is stored in the world state for easy access.

#### ServerData struct:

Server writes the encrypted parameters into the ledger. The data field in this stores the encrypted parameters, while the round stores the current FL round.

#### CurrentOrgData struct:

It is used as a metadata store for each organization.

1. **Tokens:** Current total number of avaiable tokens with the organization.
2. **RoundSeen:** This is a list of round numbers of already requested session keys. When a client requests session key of an already requested round (already requested even by any other client of the same organization), it gets the session key without the organization’s tokens getting deducted.
3. **LatestRound:** This stores the latest round in which some client of the organization wrote parameters into the ledger.

#### KeyRequests struct:

It is used to store the session key requests from different organizations. When a client requests a session key, the round, orgName of the client gets appended here if there are sufficient number of tokens. The server later queries the asset to get all the session key requests.

#### SessionKey struct:

It is used to store the session key of a particular round in the private data collection. A particular round’s session key can be accessed by the client using the round number.

The following are the chaincode functions that can be invoked:

#### Client Chaincode Functions:

1. **PutClientParams(***data, epsilon, round***):** Uploads the client’s model parameters to the blockchain ledger for the current round.
2. **GetEncryptedParams(***round***):** Fetches the encrypted global model weights from the ledger.
3. **RequestKey(***round***):** Requests the session key from the server by paying tokens, if available.
4. **GetSessionKey(***round, privateDataCollection***):** Retrieves the session key from the private data collection shared between the client’s organization and the server’s organization.

#### Server Chaincode Functions:

1. **GetAllParams(***num, seed***):** Fetches the uploaded model parameters from the ledger for the selected set of clients.
2. **SelectRandomSet(***num, seed, round***):** Selects a random subset of *num* clients to fetch the local model parameters.
3. **PutGlobalParams(***data***):** Uploads the aggregated global model weights to the ledger, after encrypting using the session key.
4. **GetRequests():** Gets the set of all clients who have requested the session key for the current round by paying tokens.
5. **PutSessionKey(***round, privateDataCollection***):** For each client that has requested the key, puts the session key into the private data collection shared between the client’s organization and the server’s organization.

### B. CLIENT AND SERVER WORKFLOW

After registration, each client initializes a pre-configured deep neural network (DNN) model equipped with randomly generated weights and biases. In each round, the client trains the model using the current model parameters and the local training data for a number of local epochs. The updated parameters are then written to the ledger by invoking the PutClient-Params (*data, epsilon, round*) function of the chaincode. This transaction is endorsed by the endorsers in accordance with the endorsement policy. Subsequently, *data* and *epsilon* are filled into the Data and Epsilon field of the OrgData asset for that client along with the *orgName* for the current round, and the LatestRound field is updated with the latest round number *round* for the CurrentOrgData asset.

Given that the system operates asynchronously, issues such as node failure or network disturbances may cause the client to fail to update local parameters and hence, its round number may fall behind. If the round number is updated as $R_{C}+1$, where $R_{C}$ is the latest round in which client C wrote param-eters to ledger, these parameters will not be considered by the server since it continues updating global parameters for several rounds and only requests the next round’s parameters from the clients. To address this, the round $R_{C}$ of the client C is updated as $\text{max}\left(R_{C}+1,\;R_{S}+1\right)$, where $R_{S}$ denotes the latest rounds in which the server wrote the parameters into the ledger. This approach ensures that even if a client remains inactive for a period of time and subsequently rejoins the network, its data is still considered during the aggregation process.

After aggregation by the server, each client is able to fetch the encrypted global parameters that have been uploaded to the ledger by the server, using the GetEncrypted-Params (*round*) function. However the client can only decrypt and read the global parameters if it possesses the session key. If the client possesses an adequate number of tokens, it can request the server for the session key using the RequestKey (*round*) function. Invoking this chaincode updates the *KeyRequests* in the ledger. The server places the session key into the private data collection shared between the client’s organization and the server’s organization, ensuring that unauthorised users cannot access the session key. Upon receiving the key, the client’s available tokens decrease by the cost of reading from the ledger. If the client requests the session key for a round it has previously requested, the cost to read that round’s session key will be zero. Now, the client fetches the key from its private data collection using the GetSessionKey (*round, privateDataCollection*) function and decrypts the global weights. The local parameters are then updated with the global model parameters. The process then repeats, starting a new round until convergence or maximum predefined rounds are reached. In each round, the server obtains the local parameters of a subset of size *num* clients by invoking the GetAllParams(*num*, *seed*) chaincode function. This function returns a random subset of *num* clients’ local parameters for the round $R_{S}+1$, where $R_{S}$ represents the server’s previous round number when it aggregated the parameters (the previous federated learning round) and wrote the global parameters to the ledger. The random subset selection of clients by the chaincode is as follows:

1. All the clients which have written their local parameters in a round $R_{S}+1$ are extracted.
2. The list of these clients is then randomly shuffled.
3. First *num* clients are selected from the list.

For each selected client, tokens are awarded as part of incentivization process based on the privacy budget ϵ. Subsequently, the server aggregates all the local model parameters off-chain. A random session key is generated for the symmetric encryption process of these aggregated parameters or the global parameters (*params*). These *params* are then encrypted with the generated session key using symmetric key encryption. These encrypted parameters are written into the ledger by invoking PutGlobalParams(*encryptedParams*).

The Server periodically invokes GetRequests() chaincode function. It returns a list of {*orgName, round*}, where orgName represents the organization which requested the session key for round. The server services their requests by invoking PutSessionKey(*round*, *privateDataCollection*) for each request, where private-DataCollection is the requested organization’s private data collection. This process happens till there are no new local parameters written into ledger.

Note that, in Steps (1)–(3) above, in each FL round, only a random fraction of the clients is selected for model update. This is done primarily for the sake of efficiency, as experiments have shown diminishing returns for adding more clients beyond a certain point [1]. Hence, in our work also, we randomly selected a subset of clients out of those that have written their local parameters in the blockchain. The reason for letting any number of clients write their weights to the ledger is primarily for ease of implementation. There are two choices - either only the randomly selected clients write their weights to the ledger, or any available clients write their weights and then the server randomly selects the clients for the current round. Since one or more clients may not be active even though they were selected, the server will have to again select their replacements. This causes synchronization difficulty especially in the blockchain environment. Hence, we choose the second option. Besides, this approach avoids having to implement a polling mechanism to notify the selected clients and then fetch their weights. Selecting the clients randomly only from those that have written their weights, circumvents both the problems. It also lets our method work when the server selects all the clients who have written their weights for the current round. Thus, the overall methodology can be executed seamlessly for both the scenarios.

## V. EMPIRICAL EVALUATION

In this section, we present the results of our empirical evaluation with the implementation mentioned in the last section. The dataset and experimental setup is first described followed by the results.

### A. MODEL ARCHITECTURE AND DATASET PREPARATION

While the proposed architecture for SBTLF is ML model and dataset agnostic, for our experiments, we have used a Convolutional Neural Network (CNN) model for image classification. The classification task considered is to identify the handwritten digits in the MNIST dataset [36], where each image is a gray scale 28 × 28pixel representation of a digit (0–9). The first layer in the model is a convolutional layer with 16 filters and a 3 × 3 convolutional kernel. Rectified Linear Unit (ReLU) activation is applied. Next, a 2 × 2 max pooling layer is employed to down-sample spatial dimensions, before passing to the third layer. This is again a convolutional layer with 32 filters, a 3 × 3 kernel and ReLU activation. Another 2 × 2 max pooling operation is applied before flattening the output from the convolutional layers into a 1D array. Then, a dense layer is added with 128 units and ReLU activation. Finally, the output layer is applied comprising 10 neurons, representing the 10 possible classes (digits 0–9). Softmax activation is employed to obtain the class probability distribution. The Adam Optimizer was chosen as the optimization algorithm, allowing adaptive learning rate modifications during training. Categorical cross entropy was used as the loss function since it is suitable for multi-class classification tasks with one-hot encoded labels. Model performance was evaluated using the accuracy metric.

### B. DETAILED RESULTS

As there are many design parameters affecting the accuracy of SBTLF, we carried out a number of experiments. In each, some of the parameters are kept fixed while varying the rest.

**FIGURE 3.**
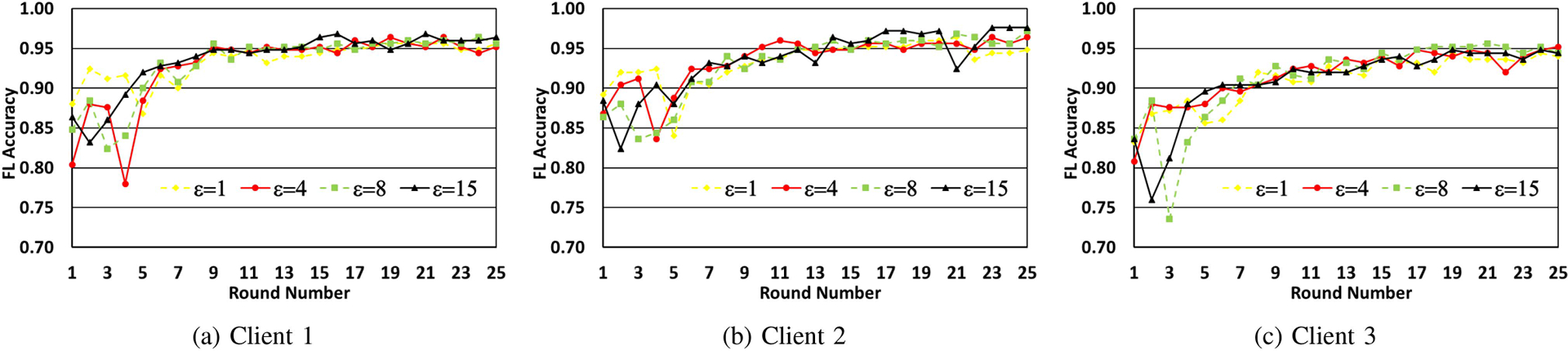
Model accuracy progression over Federated Learning rounds, for different values of epsilon. The client model is trained for 5 epochs in each round over 50 samples per digit.

In Fig. 3(a)–(c), we make each client run local training on its dataset with the current set of weights and the same value of ϵ for 5 epochs. The updated weights are written to the ledger once these epochs are completed. There are 50 samples for each digit used in training. We show the impact of variation in the number of FL rounds on accuracy for different values of the privacy parameter ϵ, for each of the clients. From the figures, it is observed that while ϵ=1 leads to poorer training, as the value of ϵ is increased, accuracy goes up significantly with the number of federated learning rounds.

**FIGURE 4.**
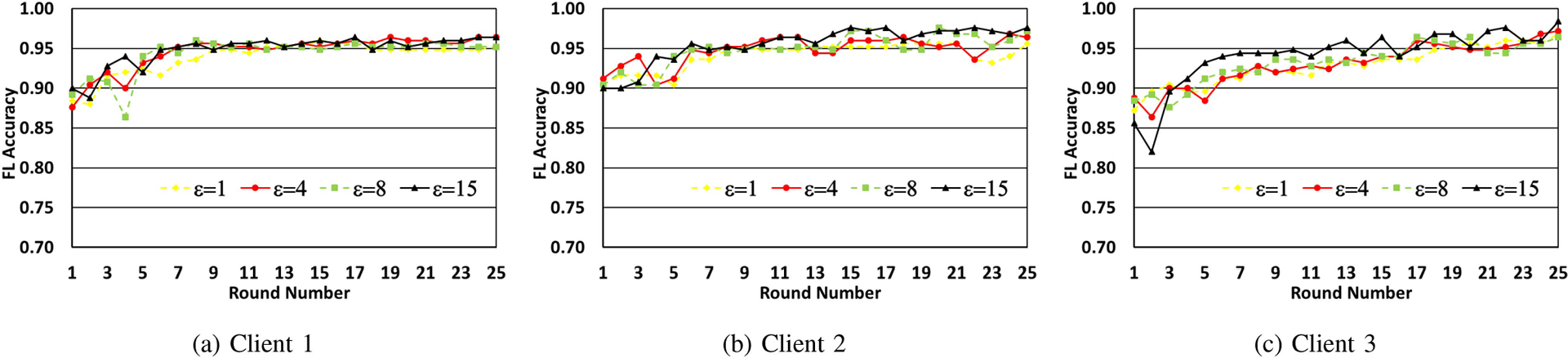
Model accuracy progression over federated learning rounds, for different values of epsilon. The client model is trained for 10 epochs in each round over 50 samples per digit.

The same experiments were next repeated by setting the number of epochs to 10 and the corresponding results are shown in Fig. 4(a)–(c). While a trend similar to that in Fig. 3(a)–(c) is seen, it is observed that for the same number of FL rounds and the value of ϵ, a higher accuracy is obtained and convergence is attained more quickly when the number of epochs used for local training is more.

**FIGURE 5.**
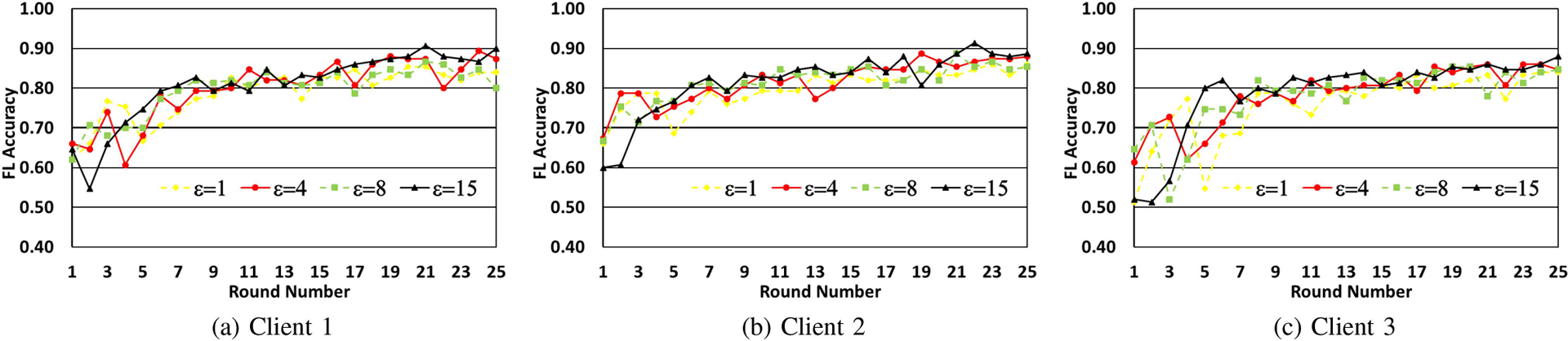
Model accuracy progression over federated learning rounds, for different values of epsilon. The client model is trained for 5 epochs in each round over 15 samples per digit.

In Figs. 5(a)–(c), we make each client train their local models for 5 epochs with 15 samples per digit. We observe a similar trend as the previous two experiments, showing that increase in ϵ increases the testing accuracy, which is as expected. We also see that the model itself does converge for lower values of ϵ even though the accuracy is a bit lower.

**FIGURE 6.**
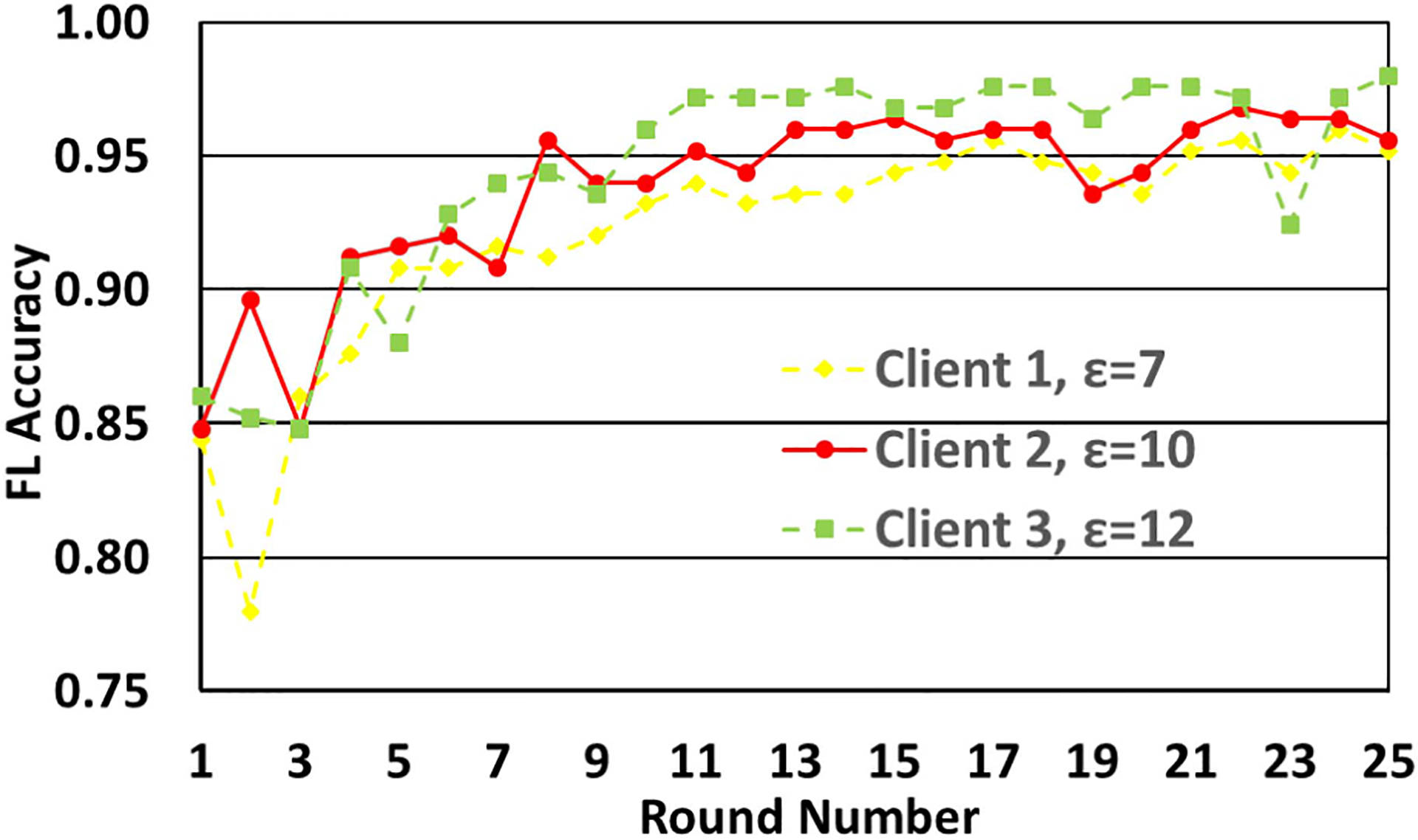
Variation of accuracy with number of FL rounds with different clients using different but fixed privacy budgets.

In the experiments so far, we used the same value of ϵ for all the clients. We next vary its value for different clients. In Fig. 6, the ϵ values are 7, 10 and 12 for Clients 1, 2 and 3, respectively. The intent is to study the impact of the absolute as well as relative variation in the value of ϵ across clients. From the plots, it is seen that ϵ has a strong impact on accuracy. As is seen from the figure, when one of the clients (Client 1) has ϵ=7, the accuracy drops significantly, since lower ϵ implies higher data obfuscation and hence higher privacy. Conversely, Client 3 having ϵ=7 has better accuracy due to less data perturbation.

**FIGURE 7.**
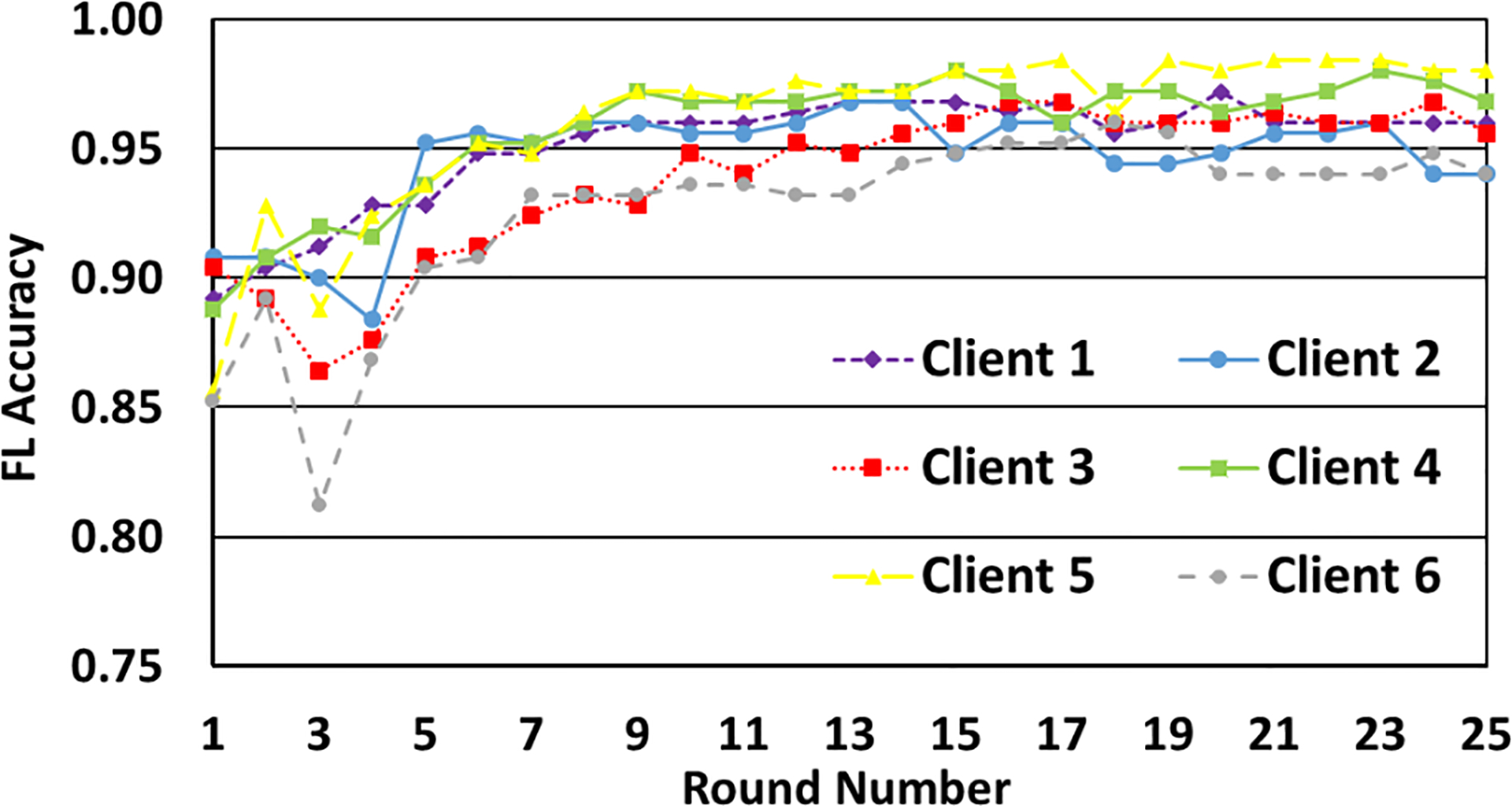
Variation of accuracy with number of FL rounds with different clients using varying privacy budgets for each round.

Finally, we run the entire setup with each client having a different value of ϵ for each round as shown in Fig. 7. This is to study the general progression of accuracy versus number of FL rounds in a real-life implementation of the system, wherein each client may wish to tailor its privacy budget after each round according to the results of the previous round. We see that even when the value of ϵ is different for different rounds, the models are converging.

**FIGURE 8.**
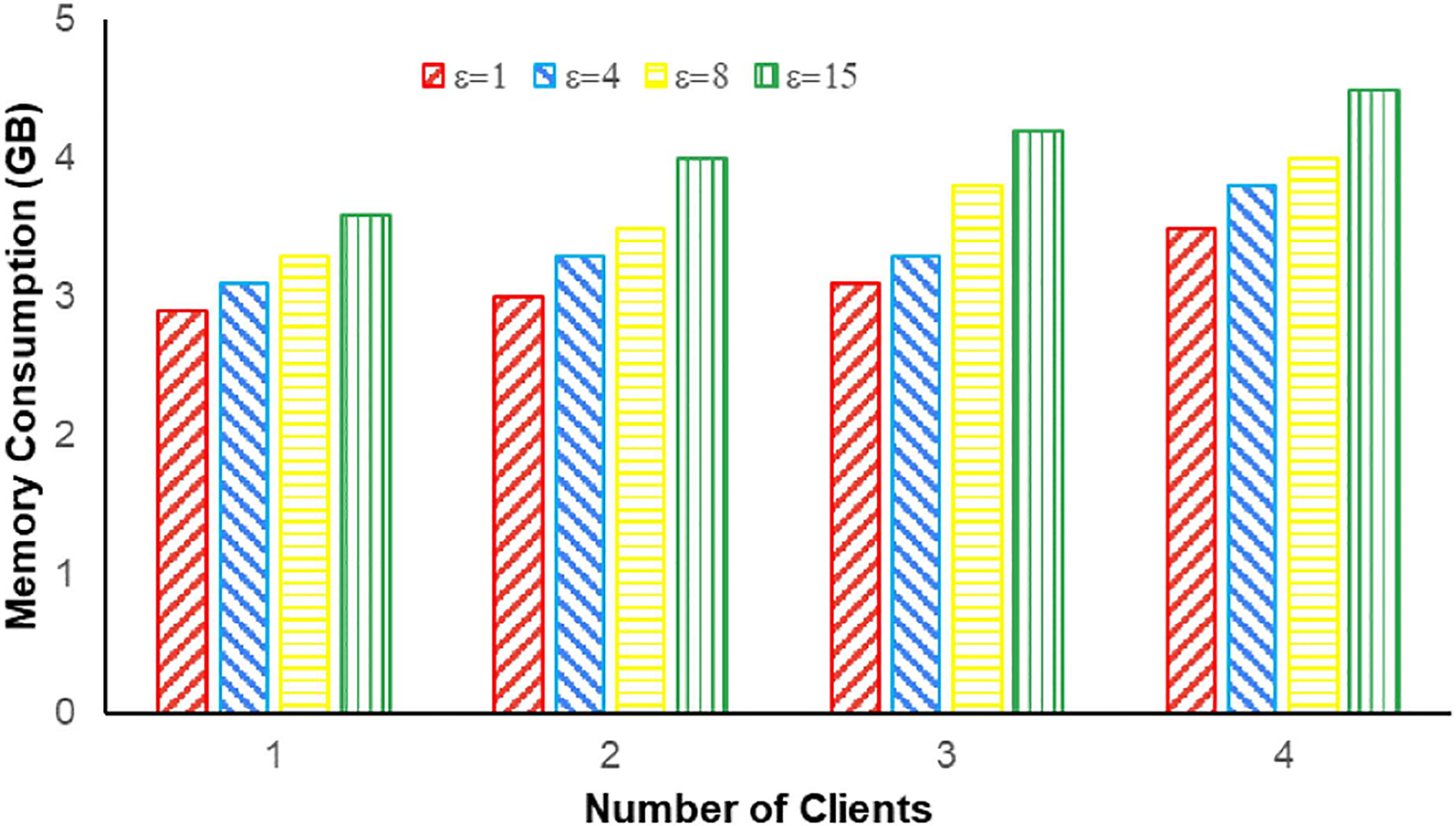
Memory consumption for different ϵ values and number of clients.

Besides demonstrating the accuracy of the complete system, we also show the scalability of the system in terms of memory consumption in Fig. 8. It is observed that the number of clients does not affect the memory consumption significantly. On the other hand, as ϵ is varied from 1 to 15, memory requirement goes up by approximately one GB. It also needs to be noted that, in an actual deployment, each node will run on a different machine. In our experiments, they have been made to run on the same machine. Thus, overall, SBTLF is highly scalable and can be effectively used to train ML models using federated learning with local differential privacy.

### C. COMPARISON WITH EXISTING WORK

There is no work in the literature that can be directly compared quantitatively with our approach. Hence, we qualitatively compare it with the state-of-the-art in Table 1. It is seen that the proposed approach has more features than any of the other comparators. Specifically, while our initial work called BTLF [18] supports most of the features, yet it cannot prevent malicious behavior of nodes from subverting the goal of incentivization. For example, if a peer node behaved maliciously, the global parameters could be accessed without any additional token costs to the clients. Also, a malicious node could deduct tokens without delivering the global parameters to the client. Further, since each peer node maintains a replicated copy of the blockchain ledger, in BTLF, they could retrieve consolidated global parameters directly from the ledger. The improved version SBTLF as presented in the current paper addresses all such concerns and can be meaningfully used by enterprises in building ML models through federated learning with LDP and appropriate incentivization.

It may be noted that, the nodes/clients themselves could possibly aggregate the individual local parameters of other nodes as the parameters are written to a ledger. This way, clients can get most recent global parameters without using its available tokens. However, we argue that if a client aggregates the local parameters of all other clients, it will need to spend its own computational power, which is more expensive than simply spending its tokens. Thus, the proposed approach discourages such behavior of clients.

## VI. RELATED WORK

Several work in the literature [16], [37], [38], [39], [46], [47], [48], [49], [50], [51] have explored decentralized federated learning by integrating FL with blockchain-based decentralized execution. While majority of these [37], [38], [47] mainly focus on secured decentralized training of the FL model through multiple clients, some [48], [50], [51] have also considered application-specific use cases for decentralized machine learning. A recent research by Hai et al. [51] introduce an innovative integration of FL and blockchain technology in developing a medical records recommendation system. Pokhrel and Chai [16] present FL with blockchain for autonomous vehicles along with automobile design challenges.

In yet another domain-specific application, Liu et al. [52] have discussed a secure FL framework for 5G networks, where both effective learning and security have a significant role. In recent years, there has been some research towards preserving privacy in federated learning. For example, Bhowmick et al. [53] introduce a method that protects against the reconstruction of parameters, thus enhancing privacy in FL. In contrast, Hao et al. [27] focus on efficiency and confidentiality in federated deep learning. In [54], the authors have developed a fully decentralized FL framework by using blockchains for implementing gradient updates. The proposed approach reduces the cost of gradient descent while prevents data and model poisoning in FL. Several other recent work [55], [56], [57], [58], [59] have attempted to ensure security in FL through blockchain-based gradient updates. In addition, application-specific approaches have been developed to ensure security in FL [60], [61]. Although they have explored secure and privacy-preserving decentralized model training through blockchain-based FL, the primary focus is on model privacy rather than data privacy for individual clients participating in the federated training procedure. LDP becomes essential in this context to ensure data privacy for the individual clients participating in the model training procedure.

While some efforts have been made to employ blockchain in the context of federated learning [15], limited research has focused on incorporating LDP in this domain. Critical design choices necessitate careful evaluation, including determining whether the same nodes perform all three crucial operations: model parameter calculation, LDP-related computation, and distributed ledger updating. Notably, in domains like the Internet of Things (IoT), where end devices may lack adequate computational power, allocating tasks to edge nodes has significant implications on device privacy. In [62], the authors have developed an incentivization mechanism over blockchain for cross-silo federated learning, where the organizations cooperatively train a global model with their local datasets, whereas some organizations may work as free-riders. The proposed approach aims at improving the social efficiency through an incentivization mechanism over the blockchain framework.

Among the various application-specific domains, Li et al. [44] consider FL for segmenting brain tumors. In contrast, Lu et al. [28] integrate differential privacy with federated learning in mobile edge computing. They emphasize the importance of privacy-preserving approaches in urban settings. Zhao et al. [30] further extended the scope of privacy preservation in federated learning by integrating blockchain technology into the mix. They specifically targeted IoT devices, providing a unique perspective on privacy challenges and solutions in this rapidly evolving domain. Likewise, the focus of the work proposed in [30] is LDP-based FL for the Internet of Things. In a more general setting, the work of Truex et al. [29] gives a formal privacy guarantee for LDP in federated learning.

Existing literature provides limited insights into the development of appropriate incentive schemes for federated learning. Kong et al. [63] propose an incentivization mechanism that does not monetize data like the other approaches. Instead, model performance is used as the reward, i.e., those making more significant contributions can access more accurate models. It has been shown that clients will benefit by sharing as much data as they possess to participate in federated learning under this incentive mechanism. Some blockchain-based FL techniques have been proposed using specific cryptocurrencies as incentives [64]. However, the potential legal implications of such currencies can impact participants’ willingness to engage. Xu et al. [26] model incentivization in federated learning with differential privacy in industrial IoT as a Stackelberg game with the aggregating server as the leader and the client nodes as followers. The server tries to maximize the utility of its available total budget by appropriately rewarding the clients for data sharing. Another line of work [65], [66] uses incentive compatibility to enable rational behavior. A systematic and comprehensive survey on incentivization in federated learning can be found in [45].

In contrast, as proposed in this paper, the token-based incentive approach SBTLF circumvents these issues and allows for seamless integration across multiple blockchain networks, directly or through appropriate conversion factors. Aligning the incentive quantum with the LDP privacy factor ϵ will foster fairness and encourage active participation in the federated learning process. However, several research challenges are associated with such an integrated approach. First, the entire process should work autonomously by executing well-developed chaincodes. While the actual local model training works off-chain, model parameter sharing, crediting, and debiting of tokens, as well as global parameter calculation and dissemination, happen on-chain. Further, the incentivization scheme must be fair and depend on the privacy budget level for the individual clients. Thus, LDP and TBI are to be designed in an integrated manner, which should also be amenable to implementation as chaincodes on a permissioned blockchain network.

## VII. CONCLUSION

We have proposed a novel approach named SBTLF for LDP-FL coupled with token-based incentivization. Our implementation of such decentralized Federated Learning within HyperLedger Fabric, a permissioned blockchain, addresses critical issues surrounding client selection fairness and server trustworthiness. By utilizing a chaincode to randomly sample a subset of nodes, we ensure that transactions are endorsed by all endorsers, adhering to the endorsement policy and there is no selection of a single client by malicious servers. Furthermore, we highlighted the asynchronous nature of the system and explained how updating the client’s round number in relation to the server’s round number and the client’s previous round number helps reduce the challenges posed by this asynchronicity to some extent.

Finally, the integration of token-based incentivization enhances the practicality of the system by encouraging clients to train their models and share data to reap the benefits of FL. Usage of symmetric key encryption to encrypt and protect global parameters ensures that only the deserving clients have access to the global model weights.

We plan to augment SBTLF by considering other LDP mechanisms as well as extending to more relaxed differential privacy models such as sensitive privacy [33], [34]. We would also like to evaluate with larger datasets that may have different data distributions. It will be interesting to consider various kinds of adversarial attacks on LDP-FL and how these can be guarded against in SBTLF. Validation of the parameters used could additionally be done as in some recent work [67].

## Figures and Tables

**TABLE 1:** Qualitative Comparison of Current and Existing Work

Related Work	LDP	FL	Blockchain	Incentivization	Data Encryption
Chakraborty *et al*. [37]	✘	✓	✓	✘	✘
Desai *et al*. [38]	✘	✓	✓	✘	✘
Issa *et al*. [39]	✘	✓	✓	✘	✘
Liu *et al*. [40]	✓	✓	✓	✘	✘
Hao *et al*. [27]	✓	✓	✘	✘	✘
Naseri *et al*. [41]	✓	✓	✘	✘	✘
Padala *et al*. [42]	✓	✓	✘	✘	✘
Triastcyn *et al*. [43]	✘	✓	✘	✘	✘
Li *et al*. [44]	✘	✓	✘	✘	✘
Zhao *et al*. [39]	✓	✓	✘	✘	✘
Witt *et al*. [45]	✘	✓	✓	✓	✘
Xu *et al*. [26]	✘	✓	✘	✓	✘
BTLF [18]	✓	✓	✓	✓	✘
SBTLF	✓	✓	✓	✓	✓

**LISTING 1:** Function Declarations of Chaincodes Invoked by Client

func PutClientParams (data string, epsilon float64, round int) {}
func GetEncryptedParams (round int) {)
func RequestKey (round int) {}
func GetSessionKey (round int, privateDataCollection string) {}

**LISTING 2:** Function Declarations of Chaincodes Invoked by Server.

func GetAllParams (num int, seed int) {)
func SelectRandomSet (num int, seed int, round int) {}
func PutGlobalParams (data string) {}
func GetRequests () {}
func PutSessionKey (round int, privateDataCollection string) {}

## Footnotes

1 LDP mechanism as mentioned in [31].
